# Obituary

**Published:** 1919-04

**Authors:** 


### OBITUARY

SIMEON H. GUILFORD, A.M., Ph.D., D.D.S., of Philadelphia, died January 15, 1919, in the Samaritan Hospital in Philadelphia. Dr. Guilford was for many years Dean of the Philadelphia Dental College, and was one of the leading educators of his time. He was greatly interested in all dental educational problems and spent much time in helping to organize the teachers of the country in the Pedagogic Institute. He was a well-educated man and a very fluent speaker. He was greatly beloved by his students, for whom he was always ready to make personal sacrifices for their encouragement. He was a most versatile instructor and was able to fill any of the chairs in his faculty when necessary. He did a prodigious amount of teaching and also kept up a large practice.

He was a prolific writer and contributor to current dental literature. He wrote a small book on nitrous oxid and another on orthodontia. He also was a contributor to the *American System of Dentistry* and the *American Text Book on Operative Dentistry.* He was in charge of the first dental unit to go to France in 1915. He has held many positions of honor in his profession which he never sought, but in which he exerted all his talent for the good of the profession. He was an indefatigable worker and it would be impossible to enumerate his contributions to the profession, to which he devoted his entire life and energies. He had a serious operation a few years ago from which he did not entirely recover.

He was a genial and wholesome man and made hosts of friends who will deeply regret his death. Few men have served the profession with such rare devotion, and his name will be recorded as one of the founders of American dentistry. He was our esteemed personal friend.

